# Combined use of CA125, neutrophil/lymphocyte ratio and platelet/lymphocyte ratio for the diagnosis of borderline and malignant epithelial ovarian tumors

**DOI:** 10.1186/s13048-023-01106-4

**Published:** 2023-02-09

**Authors:** Ke Huang, Shengjie Xu, Jiatong Wang, Lili Ge, Juan Xu, Xuemei Jia

**Affiliations:** Department of Gynecology, Women’s Hospital of Nanjing Medical University, Nanjing Maternity and Child Health Care Hospital, No.123 Mochou Road, Nanjing, 210004 People’s Republic of China

**Keywords:** Platelet/lymphocyte ratio, Neutrophil/lymphocyte ratio, Cancer antigen 125, Epithelial ovarian tumor

## Abstract

**Background:**

The mortality rate of ovarian cancer ranks first among three common gynecological malignant tumors due to insidious onset and lack of effective early diagnosis methods. Borderline epithelial ovarian tumor (BEOT) is a type of low malignant potential tumor that is typically associated with better outcomes than ovarian cancer. However, BEOTs are easily confused with benign and malignant epithelial ovarian tumors (EOTs) due to similar clinical symptoms and lack of specific tumor biomarkers and imaging examinations. Notably, a small subset of BEOTs will transform into low-grade serous ovarian carcinoma with a poor prognosis. Therefore, searching for potential biomarkers that can be easily obtained and accurately identify malignant epithelial ovarian tumors (MEOTs) as well as BEOTs is essential for the clinician. Cancer antigen 125 (CA125) is a commonly used biomarker for the diagnosis of EOTs in the preoperative scenario but has low sensitivity and specificity*.* Nowadays, inflammatory biomarkers including inflammatory cell counts and derived ratios such as neutrophil/lymphocyte ratio (NLR) and platelet/lymphocyte ratio (PLR) have been proved to be associated with tumor progression and poor prognosis, and were considered to be the most economically potential surrogate biomarkers for various malignancies. The purpose of this study was to find appropriate combinations of inflammatory and tumor biomarkers to improve the diagnostic efficiency of EOTs, especially the BEOTs.

**Results:**

CA125, NLR and PLR increased steadily among benign, borderline and malignant EOTs and tended to be higher in advanced (stage III-IV) and lymph node metastasis MEOT groups than in early stage (stage I-II) and non-lymph node metastasis MEOT groups. CA125, NLR and PLR could be used separately in the differentiation of EOTs but could not take into account both sensitivity and specificity. The combined use of CA125, NLR and PLR was evaluated to be more efficient, especially in the identification of BEOTs, with both high sensitivity and high specificity.

**Conclusions:**

The levels of CA125, NLR and PLR were closely related to the nature of EOTs and malignant progression of MEOTs. The combination of CA125, NLR and PLR was more accurate in identifying the nature of EOTs than either alone or double combination, especially for BEOTs.

**Supplementary Information:**

The online version contains supplementary material available at 10.1186/s13048-023-01106-4.

## Introduction

Ovarian cancer is one of the most common malignant tumors of female reproductive system, with the highest mortality rate among gynecological malignancies [1]. Malignant epithelial ovarian tumor (MEOT) is the most common pathological type of ovarian cancer, with insidious onset and no obvious symptoms in the early stage. In addition, there is currently a lack of early diagnosis methods for MEOT. As a result, about 75% of MEOT patients are diagnosed at an advanced stage, and the 5-year survival rate is less than 30%, while that of patients with early stage MEOT can reach more than 90% [1, 2]. At present, the discovery of simple and inexpensive biomarkers is the key to improve the early diagnosis rate and survival rate of MEOT.

Borderline epithelial ovarian tumor (BEOT) is a type of low malignant potential tumor that has better prognosis than ovarian cancer. The clinical symptoms of BEOTs in the early stage are similar to those of benign ovarian tumors, while advanced BEOTs can be complicated with abdominal distension, abdominal mass and abdominal pain, which are easily confused with ovarian cancers. Moreover, the lack of specific tumor biomarkers and imaging examinations contributes to the difficulties in the diagnosis of BEOTs and the selection of subsequent surgical methods. Additionally, it is worth noting that a small fraction of patients still experience recurrence or concealed transformation into malignant ovarian tumors, which brings great challenges to diagnosis and treatment.

Cancer antigen 125 (CA125) is the most commonly used serological marker for epithelial ovarian tumors (EOTs), especially in the follow up of women treated for high-grade serous ovarian carcinoma. However, its sensitivity is low in the preoperative evaluation. Additionally, it is also abnormally overexpressed in a variety of malignancies, including pancreatic, bladder and lung cancers, etc. [3–5] Furthermore, benign diseases such as pelvic inflammation, endometriosis and adenomyosis may also cause abnormally elevated CA125 [6]. In all, the effectiveness of CA125 was affected by its limited sensitivity and specificity.

In recently years, tumor-related inflammation theory has been widely used as a key factor in exploring tumorigenesis and tumor growth pathways [7]. Preoperative inflammatory cell counts and derived ratios such as neutrophil/lymphocyte ratio (NLR) and platelet/lymphocyte ratio (PLR), as the most direct biomarkers of the body’s inflammatory response, are related to the diagnosis and prognosis of many malignant tumors including ovarian cancer [8–15], and are considered to be the most economically potential surrogate biomarkers for various malignant tumors.

Nowadays, studies have explored the diagnostic value of tumor biomarkers combined with inflammatory biomarkers in distinguishing benign and malignant ovarian tumors [16, 17]. And the diagnosis of borderline ovarian tumors has rarely been mentioned before. The purpose of this study was to retrospectively analyze the serological indicators and pathological diagnosis of patients with EOTs, especially the BEOTs, with the aim to find a combined detection method of serological indicators to improve the diagnostic efficiency.

## Materials and methods

### Inclusion and exclusion criteria

A total of 284 patients with documented benign, borderline and malignant EOTs treated at Nanjing Maternity and Child Health Care Hospital between January 2017 and December 2020 were included in this retrospective study. Based on the postoperative pathological results reviewed by two senior pathologists, enrolled patients were divided into three groups, including 64 malignant, 64 borderline and 156 benign EOTs. Patients with MEOT did not receive chemotherapy or radiation therapy before surgery. All enrolled MEOT patients underwent comprehensive staging surgery consisting of total hysterectomy, adnexectomy, complete pelvic/para-aortic lymphadenectomy, omentectomy and peritoneal cytology. Patients with preoperative complications of blood diseases, thrombotic diseases, severe liver and kidney damage, other benign and malignant tumors, infectious diseases, autoimmune diseases, and pregnancy were excluded from the study.

### Clinical and laboratory data collection

The clinical and laboratory data included age, complete blood count, tumor biomarkers, pathological type, FIGO staging, degree of tissue differentiation, presence or absence of lymph node metastasis. The complete blood count and serum tumor biomarkers were detected before surgery within 1 week. Preoperative CA125 and HE4 concentrations were measured using a COBAS 6000 analyzer (Roche, Switzerland) with the chemiluminescent reagent kit supplied by Roche. Pathological examinations were reviewed by two senior pathologists. The collection of patients’ clinical and laboratory data was approved by the Ethics Committee of Nanjing Maternity and Child Health Care Hospital and performed in accordance with the Declaration of Helsinki.

### Statistical analysis

The statistical assessment of the data was performed using Statistical Package for Social Sciences (SPSS) for Windows 19.0 package. Continuous variables are expressed as mean ± standard deviation. Differences in baseline characteristics among the three groups were analyzed by one-way ANOVA. Differences in baseline characteristics between two groups were analyzed by T-test. The association between postoperative pathological diagnosis and CA125, NLR and PLR was analyzed by multiple Logistic regression. Sensitivity and specificity were defined by ROC curves, and differences in the area under curve (AUC) were detected by DeLong’s test using MedCalc version 20.0.3. *P* value < 0.05 was considered statistically significant.

## Results

### CA125, NLR and PLR showed significant differences among benign, borderline and malignant EOTs

A total of 284 patients were included in this study. The basic laboratory parameters of all included patients were briefly presented in Table 1. The mean ages of the patients diagnosed as benign, borderline and malignant EOTs were (48.33 ± 15.84), (47.77 ± 18.88) and (47.6 ± 20.62) years, respectively (*P* = 0.629). As shown in Table 1, the expression of CA125 showed significant differences among benign, borderline and malignant EOTs (*P* < 0.001). In addition, it was apparent that inflammatory biomarkers such as neutrophil (N), PLR, NLR and monocyte/lymphocyte ratio (MLR) showed an upward trend from benign to borderline and malignant EOTs (*P* < 0.001), while lymphocyte (L) showed the opposite trend (*P* < 0.001), suggesting that they have certain diagnostic value for benign, borderline and malignant EOTs.

**Table 1:** Laboratory parameters of participants with EOTs

Variable	Malignant	Borderline	Benign	Reference level	*P*-value
Number	64	64	156		
W (10^9^/L)	6.55 ± 3.26	5.91 ± 1.31	5.79 ± 1.91	3.5–9.5	0.0549
N (10^9^/L)	4.58 ± 3.20	3.54 ± 1.21	2.82 ± 1.42	1.8–6.3	< 0.001^abc^
L (10^9^/L)	1.51 ± 0.54	1.86 ± 0.55	2.10 ± 0.62	1.1–3.2	< 0.001^abc^
Mo (10^9^/L)	0.35 ± 0.15	0.36 ± 0.10	0.36 ± 0.13	0.1–0.6	0.868
PLT (10^9^/L)	253.8 ± 83.24	242.9 ± 59.16	189.8 ± 56.40	125–350	< 0.001^bc^
PDW (fL)	13.33 ± 2.61	13.56 ± 2.10	13.86 ± 2.95	9.6–15.2	0.5594
RDW (%)	42.63 ± 5.33	41.48 ± 2.36	42.10 ± 2.98	41.2–53.6	0.3279
PLR	193.3 ± 103.35	140.2 ± 49.61	80.61 ± 25.85	/	< 0.001^abc^
NLR	3.87 ± 6.02	2.15 ± 1.39	1.19 ± 0.47	/	< 0.001^abc^
MLR	0.27 ± 0.20	0.21 ± 0.07	0.15 ± 0.05	/	< 0.001^abc^
CA125 (U/ml)	90.57 ± 75.22	39.48 ± 47.37	12.41 ± 3.37	0–35	< 0.001^abc^
HE4 (pmol/L)	128.00 ± 186.14	58.64 ± 30.44	52.56 ± 11.01	premenopause < 70 postmenopause< 140	< 0.001^ac^

*W* white blood cell count, *N* absolute neutrophil count, *L* absolute lymphocyte count, *Mo* absolute monocyte count, *PLT* blood platelet count, *PDW* platelet distribution width, *RDW* red cell distribution width, *PLR* platelet/lymphocyte ratio, *NLR* neutrophil/lymphocyte ratio, *MLR* monocyte/lymphocyte ratio, *CA125* cancer antigen 125, *HE4* human epididymis protein 4

^a^ the difference between malignant and borderline EOTs was statistically significant

^b^ the difference between borderline and benign EOTs was statistically significant

^c^ the difference between malignant and benign EOTs was statistically significant

Compared with N, L and platelet (PLT), NLR and PLR provided a more comprehensive reflection of the inflammatory response and showed a significant gradient change, and were selected along with CA125 as indicators for follow-up studies. Considering that there was no significant difference in monocyte (Mo) among the three groups (*P* = 0.868), and MLR was essentially the reflection of differences in L among the three groups, thus no redundant studies were conducted on MLR. Additionally, human epididymis protein 4 (HE4) was excluded from subsequent studies because the difference of HE4 between borderline and benign EOTs was not statistically significant (*P* = 0.1268).

### Correlation between CA125, NLR, PLR and clinic-pathological characteristics of BEOT and MEOT patients

We further analyzed the correlation between CA125, NLR, PLR and clinic-pathological characteristics of MEOT patients. The results were shown in Table 2. Advanced MEOT (Stage III-IV) group tended to have higher CA125, NLR and PLR than early stage MEOT (Stage I-II) group. Moreover, CA125, NLR and PLR were higher in the lymph node metastasis MEOT group compared with the non-lymph node metastasis MEOT group. However, there were no statistically significant differences in CA125, NLR and PLR among different ages, histological grades and pathological types. These results suggested that higher preoperative CA125, NLR and PLR levels predict a higher probability of advanced MEOT progression and lymph node metastasis. Additionally, we also explored the levels of inflammatory and tumor biomarkers in serous and mucous BEOTs, and no statistically significant differences were found between the two groups (Supplementary data 1).

**Table 2:** Relationship between laboratory parameters and clinic-pathological characteristics of MEOT patients

Variable	N (%)	CA125(U/ml)	NLR	PLR
Age				
≤50	31 (48.4%)	99.69 ± 76.67	3.43 ± 2.45	192.2 ± 72.38
> 50	33 (51.6%)	82.00 ± 66.87	4.28 ± 7.76	194.3 ± 127.18
*P*-value		0.3636	0.5812	0.9402
FIGO staging				
I-II	41 (64.1%)	74.53 ± 65.18	2.76 ± 1.54	170.5 ± 73.25
III-IV	23 (35.9%)	119.2 ± 82.68	5.85 ± 9.50	233.9 ± 138.79
*P*-value		0.0202	0.0442	0.0194
Histological grade				
G1	36 (56.25%)	78.28 ± 69.06	2.89 ± 2.1	187.8 ± 74.88
G2-G3	28 (43.75%)	106.37 ± 79.43	5.13 ± 8.57	200.4 ± 135.94
*P*-value		0.1356	0.1345	0.6399
Pathological type				
Serous	24 (37.5%)	83.61 ± 64.07	5.01 ± 9.22	208.05 ± 144.06
Mucinous	8 (12.5%)	86.93 ± 70.38	4.52 ± 3.73	200.92 ± 66.65
Endometrioid	14 (21.9%)	120.44 ± 104.28	3.41 ± 2.22	181.56 ± 61.67
Clear cell	18 (28.1%)	78.24 ± 60.69	2.42 ± 1.06	179.38 ± 87.07
*P*-value		0.403	0.551	0.807
Lymph nodes				
Negative	51 (79.7%)	72.88 ± 8.93	2.69 ± 0.20	175.6 ± 10.37
Positive	13 (20.3%)	159.97 ± 18.11	8.50 ± 3.36	262.7 ± 47.05
*P*-value		< 0.0001	0.0012	0.0067

### Efficiency of single use of CA125, NLR and PLR in diagnosing benign, borderline and malignant EOTs

**Fig. 1:**
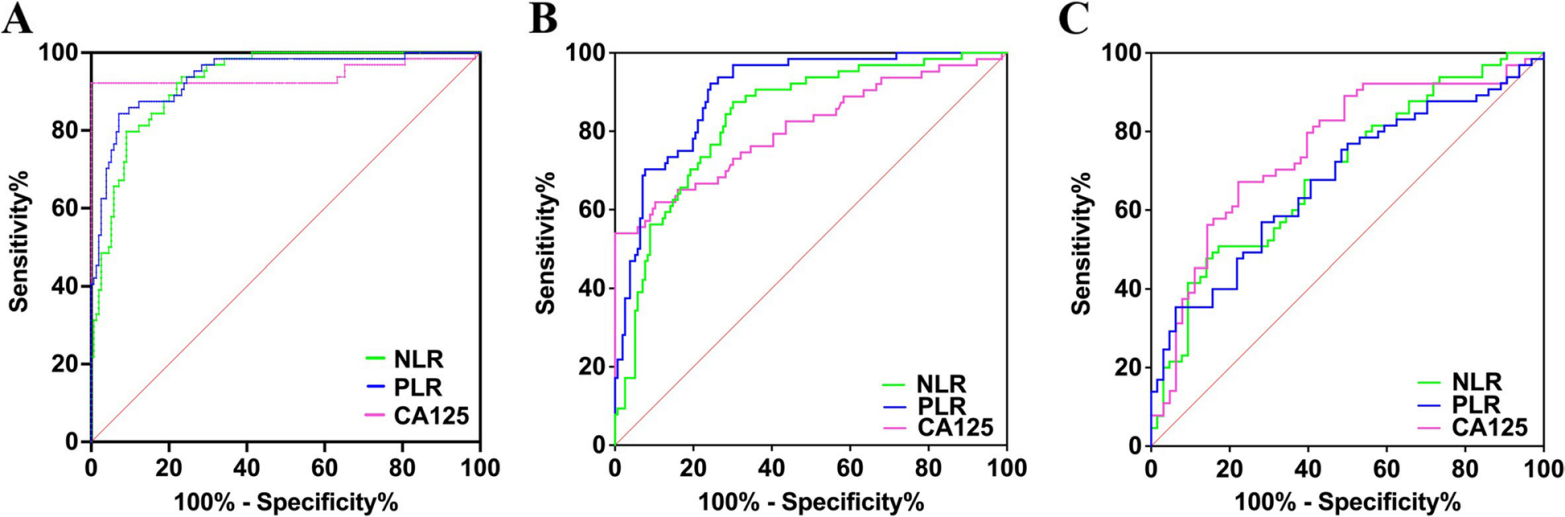
ROC curves of CA125, NLR and PLR in the diagnosis of benign, borderline and malignant EOTs. **A** benign vs. malignant **B** benign vs. borderline. **C** borderline vs. malignant

According to the association of CA125, NLR and PLR with EOTs, ROC curves were made and used to determine the optimal cut-off value and the corresponding sensitivity and specificity (Fig. 1A, B, C). The results were shown in Table 3. The cut-off value of CA125 in distinguishing benign and malignant EOTs was selected according to the commonly used clinical value, namely CA125 = 35 U/ml. When Youden Index reached the maximum, the optimal cut-off values of NLR and PLR for distinguishing benign and malignant EOTs were 1.76 and 114.3. The corresponding sensitivity were 70.31, 79.69 and 84.38%, and the corresponding specificity were 100, 90.97 and 92.9%, respectively. When it came to identify benign and borderline EOTs, the optimal cut-off values of CA125, NLR and PLR were 18.72, 1.244 and 89, respectively. The corresponding sensitivity were 53.97, 87.5 and 92.06%, and the corresponding specificity were 100, 69.87 and 75.48%, respectively. Apart from this, when differentiating borderline and malignant EOTs, the optimal cut-off values of CA125, NLR and PLR were 42.74, 2.687 and 213.6, respectively. The corresponding sensitivity were 67.19, 46.88 and 34.85%, and the corresponding specificity were 77.78, 85.71 and 93.75%, respectively.

**Table 3:** Cut-off value and diagnostic value of CA125, NLR and PLR in the diagnosis of EOTs

Variable	Groups	AUC	Cut-off	*P*-value	Sensitivity (%)	Specificity (%)	Comparison of AUC	
							Groups	*P*-value
Benign vs. malignant	CA125	0.9417	35	< 0.0001	70.31	100	a vs.b	0.5948
	NLR	0.9271	1.760	< 0.0001	79.69	90.97	b vs. c	0.5645
	PLR	0.9399	114.3	< 0.0001	84.38	92.9	a vs. c	0.9506
Benign vs. borderline	CA125	0.8069	18.72	< 0.0001	53.97	100	a vs. b	0.5600
	NLR	0.8345	1.244	< 0.0001	87.5	69.87	b vs. c	0.0359
	PLR	0.8993	89	< 0.0001	92.06	75.48	a vs. c	0.0142
Borderline vs. malignant	CA125	0.7557	42.74	< 0.0001	67.19	77.78	a vs. b	0.2115
	NLR	0.6942	2.687	0.0001	46.88	85.71	b vs. c	0.6678
	PLR	0.666	213.6	0.0006	34.85	93.75	a vs. c	0.1265

^a ^CA125

^b ^NLR

^c ^PLR

In general, CA125, NLR and PLR could be used to predict and differentiate benign, borderline and malignant EOTs, especially in the differentiation between benign and malignant tumors. However, the ability to simultaneously ensure both sensitivity and specificity in identifying BEOTs was not ideal.

### Efficiency of CA125 combined with NLR and/or PLR in diagnosing benign, borderline and malignant EOTs

**Fig. 2:**
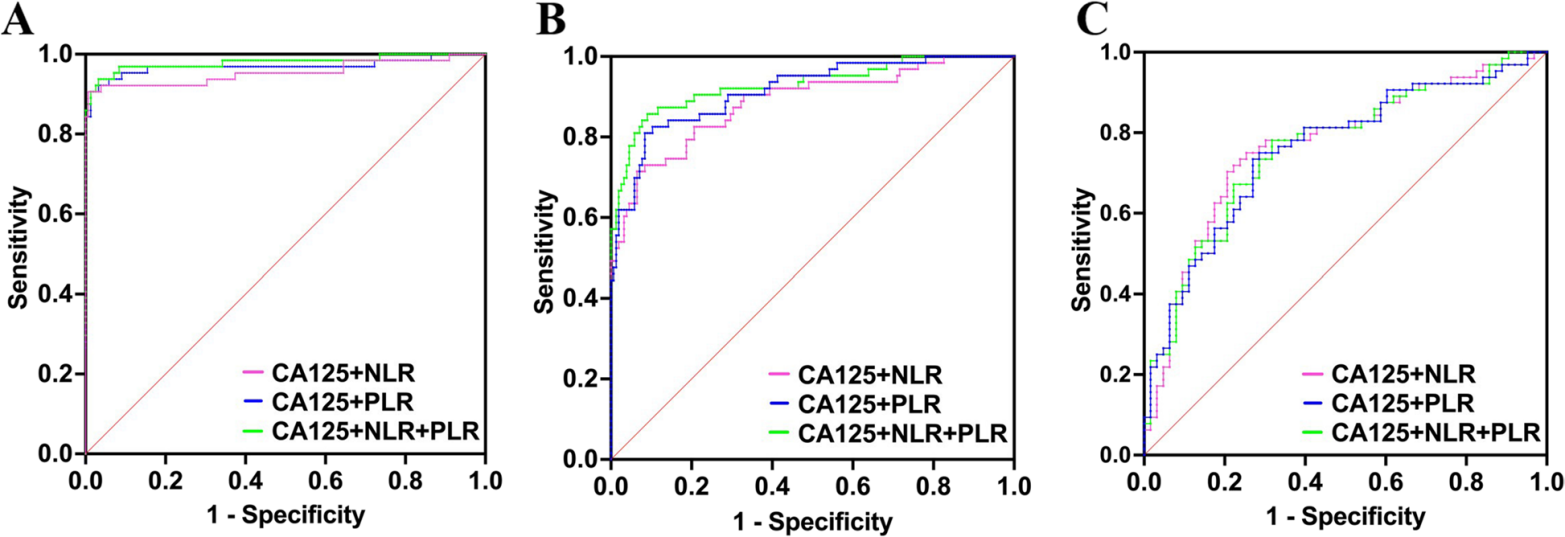
ROC curves of CA125 combined with NLR and/or PLR in the diagnosis of benign, borderline and malignant EOTs. **A** benign vs. malignant **B **benign vs. borderline. **C** borderline vs. malignant

By constructing multiple Logistic regression model, the diagnostic system of CA125 combined with NLR and/or PLR was evaluated in Table 4. Figure 2A showed the ROC curves of CA125 combined with NLR and/or PLR to identify benign and malignant EOTs. The sensitivity and specificity of CA125 combined with NLR to differentiate benign from malignant EOTs were 90.63 and 99.35%, respectively, while that of CA125 combined with PLR were 90.63 and 98.71%, respectively. In addition, when combining three indicators to identify benign and malignant EOTs, the sensitivity and specificity were 93.75 and 96.77%, respectively. Figure 2B showed the ROC curves of CA125 combined with NLR and/or PLR to identify benign and borderline EOTs. The sensitivity and specificity of CA125 combined with NLR to differentiate benign from borderline EOTs were 71.43 and 93.55%, respectively, while that of CA125 combined with PLR were 80.95 and 91.61%, respectively. In addition, the sensitivity and specificity of the three indicators combined to identify benign and borderline EOTs were 85.71 and 90.97%, respectively. The ROC curves of CA125 combined with NLR and/or PLR to discriminate between borderline and malignant EOTs were shown in Fig. 2C. The sensitivity and specificity of CA125 combined with NLR were 70.31 and 79.37%, respectively, and that of CA125 combined with PLR were 73.44 and 73.02%, respectively. Additionally, the sensitivity and specificity of the three indicators combined were 78.13 and 68.25%, respectively. Overall, CA125 combined with NLR and/or PLR made up for the shortcomings of single use, especially in the identification of BEOTs, ensuring high sensitivity and specificity. According to the sensitivity and specificity as well as differences in AUC, the combination of CA125, NLR and PLR had better application value than CA125 combined with NLR or PLR (Table 4), and was further superior to the application of CA125, NLR and PLR alone (Supplementary data 2).

**Table 4:** Diagnostic value of CA125 combined with NLR and/or PLR in the diagnosis of EOTs

Variable	Groups	AUC	Cut-off	*P*-value	Sensitivity (%)	Specificity (%)	Comparison of AUC	
							Groups	*P*-value
Benign vs. malignant	CA125 + NLR	0.9540	0.3679	< 0.0001	90.63	99.35	a vs. b	0.1785
	CA125 + PLR	0.9692	0.4404	< 0.0001	90.63	98.71	b vs. c	0.1217
	CA125 + NLR + PLR	0.9793	0.2401	< 0.0001	93.75	96.77	a vs. c	0.0491
Benign vs. borderline	CA125 + NLR	0.8856	0.3132	< 0.0001	71.43	93.55	a vs. b	0.0932
	CA125 + PLR	0.9144	0.2715	< 0.0001	80.95	91.61	b vs. c	0.1981
	CA125 + NLR + PLR	0.9287	0.2699	< 0.0001	85.71	90.97	a vs. c	0.0037
Borderline vs. malignant	CA125 + NLR	0.7676	0.4350	< 0.0001	70.31	79.37	a vs. b	0.6527
	CA125 + PLR	0.7584	0.4406	< 0.0001	73.44	73.02	b vs. c	0.5866
	CA125 + NLR + PLR	0.7617	0.4129	< 0.0001	78.13	68.25	a vs. c	0.7360

^a ^CA125+NLR

^b ^CA125+PLR

^c ^CA125+NLR+PLR

## Discussion

CA125, detected by Bast, et al. [18] in 1981 from MEOT antigen, does not exist in normal ovarian tissue and is the most classic tumor biomarker for the diagnosis of EOTs used in clinical practice. However, CA125 is also elevated in varieties of malignancies and several benign diseases. With the development of tumor pathogenesis, the relationship between inflammation and malignancy has been widely recognized. Current studies suggested that the presence of inflammation maintains the microenvironment in which malignancy occurs and progresses. In turn, malignant tumors are accompanied by inflammatory response, producing inflammatory cytokines that promote tumor progression through multiple signaling pathways. In the case of MEOT, factors associated with epithelial ovarian inflammation, such as ovulation, endometriosis and pelvic inflammatory disease, all increase the risk of MEOT [19–21]. The exchange of ascites and stroma in MEOT is accompanied by changes in lymphocyte population and their related factors, which promote the growth and spread of cancer cells [22, 23]. Therefore, inflammation and malignancy interact and promote each other.

BEOT is a type of ovarian tumor with low malignant potential. How to accurately diagnose and treat BEOTs is a great challenge for clinicians in clinical practice, which brings a series of problems. Early symptoms of BEOTs are always similar to benign ovarian tumors. When BEOTs are misdiagnosed as benign EOTs for long-term follow-up, there is a risk of secondary malignant lesions and delayed treatment. BEOTs incidentally found during operation will lead to temporary extension of the surgical scope and bring hidden danger to clinical safety. Additionally, if BEOTs are mistaken for MEOTs due to insufficient preoperative evaluation, it will lead to unnecessary expansion of the scope of surgery and increase surgical damage to patients without clinical benefit. So the diagnosis of BEOTs, especially the distinguishing of BEOTs from benign and malignant EOTs is also worthy of attention. At present, there are few studies on the correlation between BEOTs and inflammatory biomarkers, and even fewer studies have addressed the diagnostic application of inflammatory biomarkers in BEOTs.

In this study, we retrospectively analyzed the correlation between laboratory parameters and clinic-pathological characteristics of 284 EOTs. Consistent with previous studies [14–17], we confirmed that the levels of inflammatory biomarkers such as NLR and PLR were potential biomarkers in EOTs, either alone could be applied to distinguish benign and malignant EOTs and had certain diagnostic efficiency. And the combination of CA125, NLR and PLR generally balanced the sensitivity and specificity. Additionally, we innovatively explored the value of inflammatory biomarkers in the identification of BEOTs. We noticed that PLR showed significant diagnostic accuracy in distinguishing BEOTs from benign EOTs, and the triple combination of CA125, NLR and PLR optimized the diagnostic efficiency compared with single use or double combination. Our study contributes to the clinical management decisions of the clinician about indistinguishable benign and borderline EOTs.

In clinical practice, another main challenge is to differentiate women with BEOTs from those with MEOTs. Unfortunately, our study showed that adding inflammatory biomarkers to CA125 did not achieve the satisfactory efficiency (sensitivity and specificity~ 70%) in distinguishing BEOTs from MEOTs. For now, preoperative biomarkers can help but management may still depend mainly on imaging examinations followed the abnormal results of these serum biomarkers. Therefore, it is necessary to expand the sample size of BEOTs and MEOTs to evaluate this diagnostic system more accurately in the future. And with the in-depth exploration of tumor-related inflammatory mechanisms, more appropriate combinations of inflammatory and tumor biomarkers are expected to be found.

## Conclusions

To sum up, NLR and PLR are new biological indicators for the diagnosis and evaluation of EOTs, especially for BEOTs. Considering that PLR may be less affected by infections and autoimmune diseases, the diagnostic value of PLR must be appreciated. Due to high rate of false positives, CA125 assessment by adding NLR and PLR could help make the correct diagnosis preoperatively. This combined diagnostic system is inexpensive, easy to access and operate, making it suitable for clinical promotion.

## Supplementary Information

**Additional file 1: Supplementary data 1.** No significant differences in laboratory parameters between serous and mucinous BEOT patients.

**Additional file 2: Supplementary data 2.** Comparison of AUC of single and combined use of CA125, NLR and PLR.

## Data Availability

The datasets generated and/or analyzed during the current study are not publicly available due to the need for subsequent studies but are available from the corresponding author on reasonable request.
