# Supplementary material for: Combined use of CA125, neutrophil/lymphocyte ratio and platelet/lymphocyte ratio for the diagnosis of borderline and malignant epithelial ovarian tumors
Source: J Ovarian Res. 2023 Feb 9;16:37. doi: 10.1186/s13048-023-01106-4 (PMC9912622; doi:10.1186/s13048-023-01106-4)
Supplement: Supplementary file 1 — Additional file 1: Supplementary data 1. No significant differences in laboratory parameters between serous and mucinous BEOT patients. [file 13048_2023_1106_MOESM1_ESM.docx]

**Supplementary data 1** No significant differences in laboratory parameters between serous and mucinous BEOT patients

| Variable | Serous | Mucinous | Reference level | *P*-value |
| --- | --- | --- | --- | --- |
| Number | 31 | 30 |  |  |
| W (10^9^/L) | 5.78±1.16 | 6.05±1.53 | 3.5-9.5 | 0.4349 |
| N (10^9^/L) | 3.41 ± 1.09 | 3.65±1.37 | 1.8-6.3 | 0.4589 |
| L (10^9^/L) | 1.88 ± 0.60 | 1.85±0.51 | 1.1-3.2 | 0.8476 |
| Mo (10^9^/L) | 0.35±0.10 | 0.37 ± 0.11 | 0.1-0.6 | 0.6242 |
| PLT (10^9^/L) | 247.4 ± 51.11 | 238.3±69.16 | 125-350 | 0.5608 |
| PDW (fL) | 13.48±2.22 | 14.05±2.02 | 9.6-15.2 | 0.3078 |
| RDW (%) | 41.76±4.61 | 41.75±2.62 | 41.2-53.6 | 0.9880 |
| PLR | 143.9±53.42 | 134.5±45.40 | / | 0.4634 |
| NLR | 2.07±1.22 | 2.20±1.58 | / | 0.7245 |
| MLR | 0.20±0.06 | 0.21±0.08 | / | 0.6406 |
| CA125 (U/ml) | 42.86±49.27 | 37.44±46.73 | 0-35 | 0.6611 |
| HE4  (pmol/L) | 58.91±34.87 | 58.81±26.72 | premenopause <70  postmenopause<140 | 0.9899 |
