# Supplementary material for: Combined use of CA125, neutrophil/lymphocyte ratio and platelet/lymphocyte ratio for the diagnosis of borderline and malignant epithelial ovarian tumors
Source: J Ovarian Res. 2023 Feb 9;16:37. doi: 10.1186/s13048-023-01106-4 (PMC9912622; doi:10.1186/s13048-023-01106-4)
Supplement: Supplementary file 2 — Additional file 2: Supplementary data 2. Comparison of AUC of single and combined use of CA125, NLR and PLR. [file 13048_2023_1106_MOESM2_ESM.docx]

**Supplementary data 2** Comparison of AUC of single and combined use of CA125, NLR and PLR

| Variable | Groups | *P*-value |
| --- | --- | --- |
| Benign vs.  malignant | CA125+NLR+PLR vs. CA125 | 0.0383 |
|  | CA125+NLR+PLR vs. NLR | 0.0055 |
|  | CA125+NLR+PLR vs. PLR | 0.0371 |
| Benign vs. borderline | CA125+NLR+PLR vs. CA125 | 0.0001 |
|  | CA125+NLR+PLR vs. NLR | 0.0002 |
|  | CA125+NLR+PLR vs. PLR | 0.1040 |
| Borderline vs. malignant | CA125+NLR+PLR vs. CA125 | 0.8144 |
|  | CA125+NLR+PLR vs. NLR | 0.0762 |
|  | CA125+NLR+PLR vs. PLR | 0.0136 |
